# Prediction of motor function in patients with traumatic brain injury using genetic algorithms modified back propagation neural network: a data-based study

**DOI:** 10.3389/fnins.2022.1031712

**Published:** 2023-01-19

**Authors:** Hui Dang, Wenlong Su, Zhiqing Tang, Shouwei Yue, Hao Zhang

**Affiliations:** ^1^Cheeloo College of Medicine, Shandong University, Jinan, Shandong, China; ^2^China Rehabilitation Research Center, Beijing, China; ^3^School of Health and Life Sciences, University of Health and Rehabilitation Sciences, Qingdao, Shandong, China

**Keywords:** TBI, back propagation neural network, prediction model, FMA, database

## Abstract

**Objective:**

Traumatic brain injury (TBI) is one of the leading causes of death and disability worldwide. In this study, the characteristics of the patients, who were admitted to the China Rehabilitation Research Center, were elucidated in the TBI database, and a prediction model based on the Fugl-Meyer assessment scale (FMA) was established using this database.

**Methods:**

A retrospective analysis of 463 TBI patients, who were hospitalized from June 2016 to June 2020, was performed. The data of the patients used for this study included the age and gender of the patients, course of TBI, complications, and concurrent dysfunctions, which were assessed using FMA and other measures. The information was collected at the time of admission to the hospital and 1 month after hospitalization. After 1 month, a prediction model, based on the correlation analyses and a 1-layer genetic algorithms modified back propagation (GA-BP) neural network with 175 patients, was established to predict the FMA. The correlations between the predicted and actual values of 58 patients (prediction set) were described.

**Results:**

Most of the TBI patients, included in this study, had severe conditions (70%). The main causes of the TBI were car accidents (56.59%), while the most common complication and dysfunctions were hydrocephalus (46.44%) and cognitive and motor dysfunction (65.23 and 63.50%), respectively. A total of 233 patients were used in the prediction model, studying the 11 prognostic factors, such as gender, course of the disease, epilepsy, and hydrocephalus. The correlation between the predicted and the actual value of 58 patients was *R*^2^ = 0.95.

**Conclusion:**

The genetic algorithms modified back propagation neural network can predict motor function in patients with traumatic brain injury, which can be used as a reference for risk and prognosis assessment and guide clinical decision-making.

## 1. Introduction

Traumatic brain injury (TBI) is not a one-time event but a condition, which is one of the leading causes of death and disability worldwide. This disease can develop among people of any age, often causing labor loss (Brain and Spinal Injury Center, 2021). With the development of medicine, the survival rate of patients, suffering from multi-degree TBI, increased and the number of disabilities in patients decreased, improving the quality of patients’ life and their social issues (Pickelsimer et al., 2006). It is essential to predict the functional outcome, which improves the therapeutic schedule and adjusts personal livelihood (Yu et al., 2015).

In the previous studies, which predicted the mortality and GCS score, the indicators, such as GCS score, pupil response, and laboratory examination, were mainly identified in the acute phase after trauma (Roberts et al., 2004; Hukkelhoven et al., 2005; Perel et al., 2006, 2008; Rogers et al., 2010; Subaiya et al., 2012; Ercole et al., 2021). As compared to the patients in the acute phase, those in the chronic phase tended to be stable with decreased cerebral edema, and fewer acute intracranial lesions, which were favorable factors for the prediction of prognosis. However, many factors, such as rehabilitation therapies, location of the lesion, limb complications, etc., might affect the outcome of motor function.

As dysfunction is a common outcome of moderate to severe TBI. The alleviation of dysfunction and improvement of the quality of life are the primary research focus. The indicators, including GCS and pupil response, are not suitable for the prediction of outcomes in the chronic phase. Therefore, the degree of dysfunction in the patients with TBI, especially the motor function, is needed to be predicted (Perel et al., 2008; Reith et al., 2017). In the current clinical practices, the rehabilitation assessment, such as the functional independence measure (FIM), which assesses the overall prognosis of living ability, and the Fugl-Myer assessment scale (FMA), which assesses the motor function, might be used to cope with this deficiency (Ottenbacher et al., 1996; Segal et al., 1996; Zarshenas et al., 2019). The current study focused on the FMA and its effects on the upper and lower limbs.

Corticosteroid Randomization After Significant Head Injury (CRASH) and International Traumatic Brain Injury Clinical Trials Prognosis and Analysis Task (IMPACT) are relatively mature models, which can predict the outcome of TBI; their prognostic potential has been evaluated (Boyd et al., 1987; Dodds et al., 1993; Edwards et al., 2005; Rogers et al., 2010; Ercole et al., 2021). The foundation and validation of the prediction model are based on TBI clinical trials, including Collaborative European Neurotrauma Effectiveness Research in TBI (CENTER-TBI), Translational Research, and Clinical Knowledge in TBI (TRACK-TBI), and Japan Neurotrauma Database (JNTDB) (Steyerberg et al., 2008; Maeda et al., 2019; Gao et al., 2020). Based on the available data, the prediction models were built using statistical models, such as correlation, regression, non-linear fitting, and artificial neural networks (ANN) analyses. Although the previously established model could not predict FMA in the patients with dysfunction after TBI, these methods can be used to establish a prediction model.

The backpropagation (BP) neural model is one of the most common and mature models based on ANN. A large number of studies have used ANN to predict the prognosis and complications of TBI. However, these models focus on the outcome prediction, such as mortality, rather than the function prediction. This study aimed to use the BP neural network model for predicting the FMA outcome in TBI patients in the chronic phase.

## 2. Methodology

### 2.1. Research design

In this study, the measures were retrospectively analyzed at the time of the patient’s admission to the Beijing Boai hospital and during hospitalization. The possible indicators, which were related to the motor function of the patients, were also analyzed. The demographic information of the patients, excluding their personal information, was collected. The study was approved by the Medical Ethics Committee of China Rehabilitation Research Center (No. 2021-026-1).

### 2.2. Data source and population

A total of 463 TBI patients, who were admitted to the China Rehabilitation Research Center from June 2016 to June 2020, were enrolled in this study. According to the International Statistical Classification of Diseases and Related Health Problems-tenth edition (ICD-10),the diagnosis of the hospitalized patients in this study included TBI (S06.902), traumatic intracranial hematoma (S06.806), diffuse axonal injury (S06.204), concussion (S06.001), subdural hematoma (S06.501), epidural hematoma (S06.401), traumatic subarachnoid hemorrhage (S06.601), and brain contusion and laceration (S06.201).

The criteria for the inclusion of patients in this included (1) definitive diagnosis of TBI; (2) complete availability of required data; and (3) the patients performed physical and occupying therapy during hospitalization. The criteria for the exclusion of patients from this included (1) the patients with the disorders of consciousness; (2) the patients in unstable periods; (3) the patients with dyskinesia caused by fractures or any other reason; (4) the patients, who were hospitalized for less than 4 weeks; and (5) the patients, who did not cooperate due to other reasons.

### 2.3. Data preparation

In this study, the data of the patient’s age, gender, course of TBI, years of education, marital status, occupation, place of residence (urban or rural), history of tobacco and alcohol use, medical history, injury location, cause of injury, lesion laterality, type of surgery, skull defect, clinical symptoms, diagnosis, tracheotomy, complications, such as epilepsy, hydrocephalus and aphasia, mental or emotional disorders, motor dysfunction, such as shoulder subluxation, shoulder-hand syndrome, disuse syndrome, and joint spasm, and rehabilitation therapies, etc., was collected. The measures, including Fugl-Meyer balance scale (FMB), Hand Practicability assessment scale, Mini-Mental State Examination Scale (MMSE), and Fugl-Meyer assessment scale (FMA), were calculated at the time of the patient’s admission to the hospital, and the FMA scores were calculated after 4 weeks of admission.

Data scoring: TBI was given scores, ranging from 1 to 5 for <1 month, 1–3 months, 3–6 months, 4 months–1 years, and >1 year, respectively. The male and female patients were indicated by 0 and 1, respectively. The presence and absence of other complications or treatments were indicated by 1 and 0, respectively. Pearson correlation was performed using SPSS 25 for determining the factors related to the outcome (4 weeks after admission), which were also included in the establishment of the prognosis model. For each input and output item, the score was recorded as P_(initial)_, while the highest score was recorded as P_(maximum)_. The score of each patient was calculated as P = P_(initial)_/P_(maximum)_. The scores of all items were used as input in the predicting model.

### 2.4. Data analysis and model construction

The correlation analysis showed that the factors, including gender, course of TBI, rehabilitation treatment, epilepsy or hydrocephalus, aphasia, tracheotomy, concurrent dysfunction, balance, and motor function, were significantly correlated with F1 (Table 1).

**TABLE 1:** The information of predictive factors.

	Overall (*n* = 233)	Male (*n* = 171)	Female (*n* = 62)
Course of disease (d)			
<30	11 (4.72%)	7 (4.09%)	4 (6.45%)
30∼90	68 (29.18%)	55 (32.16%)	13 (20.97%)
90∼180	70 (30.04%)	49 (28.65%)	21 (33.87%)
180∼365	45 (19.31%)	27 (15.79%)	18 (29.03%)
>365	39 (16.74%)	33 (19.30%)	6 (9.68%)
**Complications and** **dysfunctions**			
Hydrocephalus	85 (36.48%)	60 (35.09%)	25 (40.32%)
Epilepsy	57 (24.46%)	47 (27.49%)	10 (16.13%)
Aphasia	110 (47.21%)	78 (45.61%)	32 (51.61%)
Other*	82 (35.19%)	52 (30.41%)	30 (48.39%)
**Treatment**			
Tracheotomy	147 (63.09%)	107 (62.57%)	40 (64.52%)
Rehabilitation therapy	182 (78.11%)	128 (74.85%)	54 (87.10%)
**Admission assessment**			
FMA	57.59 ± 33.94	60.12 ± 33.86	50.63 ± 33.47
FMB	6.67 ± 4.75	6.86 ± 4.71	6.12 ± 4.87

*Other, including disuse syndrome, shoulder subluxation or shoulder-hand syndrome; FMA, Fugl-Myer assessment scale; FMB, balance subscale of the Fugl-Meyer test.

**FIGURE 1:**
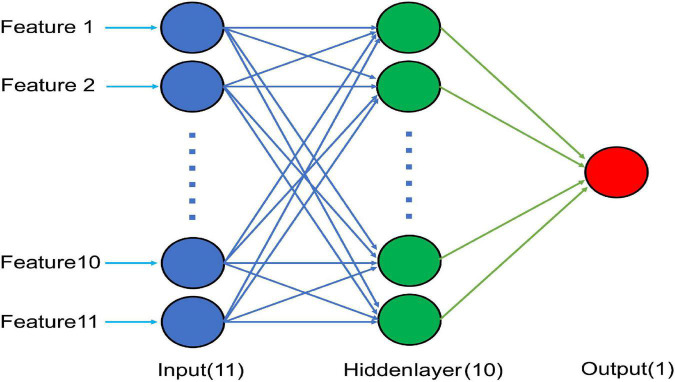
Schematic diagram of the optimal BP neural network model with 11 input nodes, 10 nodes in a hidden layer, and a single output node.

This study used the neural fitting application in MATLAB 2021b software. The GA-BP neural model was applied to predict the motor function. A one-layer neural network model (Figure 1) and FMA were applied for the normalization of input and output data, respectively. To prevent over-fitting and realize external inspection, authors divided patients into two subgroups, one with 175 (75%) patients to construct and test the model, the other with 58 (25%) patients to inspect the model. The correlations between the predicted and actual values were analyzed. Then, the prediction effects were verified and the prediction differences were calculated (mapping software origin 9.1).

## 3. Results

**FIGURE 2:**
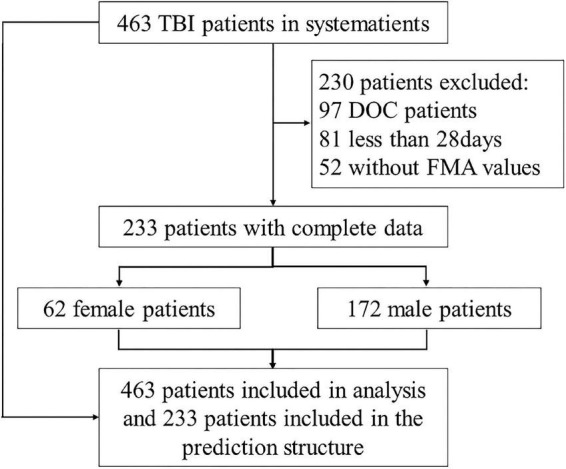
Research data source and population. DOC, disturbance of consciousness.

This study included a total of 463 patients (Supplementary material). Among them, a total of 233 patients were included in the analysis of the neural network model (Figure 2).

### 3.1. General information

Among the patients included in this study, males were significantly more than females (348:115) but did not show significant differences in their ages, courses after TBI, and durations of hospital stay. However, the proportion of smoking and drinking among males was significantly higher as compared to the females (χ^2^ = 33.62, *P* < 0.01, OR = 6.22). Meanwhile, the incidences of concurrent diseases among males, such as hypertension, diabetes, coronary heart disease, and cerebrovascular accident were significantly higher as compared to those among the females (χ^2^ = 9.41, *P* < 0.01, OR = 1.96).

The causes of injuries among males were relatively diverse, among which, the car accident was the major cause, accounting for 47.41% of the causes of injuries. The females showed similar results; car accidents accounted for 84.35% of the causes of injuries, followed by falls (15.65%).

**FIGURE 3:**
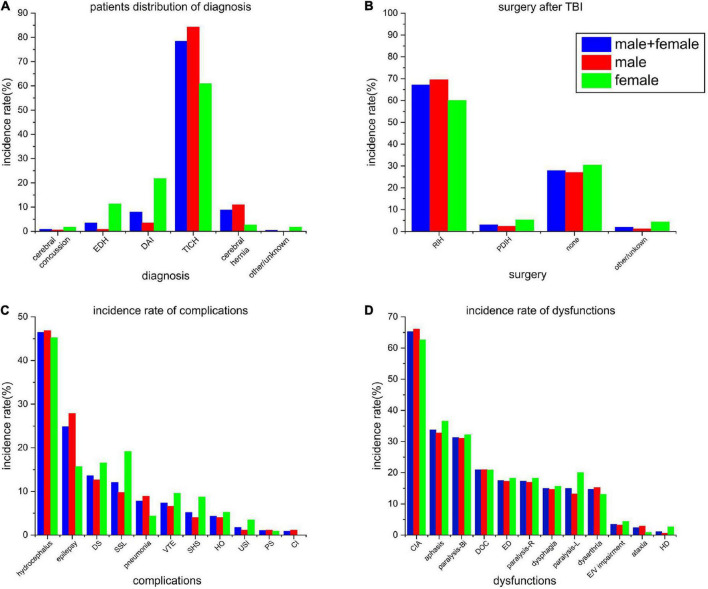
**(A)** Patient distribution of incidence rate of principal diagnosis. **(B)** Rate of the different types of surgeries after TBI. **(C)** Incidence rates of all the complications at the time of admission. **(D)** Incidence rate of all the dysfunctions at the time of admission. EDH, epidural hemorrhage; DAI, diffuse axonal injury; TICH, traumatic intracranial hemorrhage; RIH, removal of intracranial hemorrhage; PDIH, puncture drainage of intracranial hemorrhage; DS, disuse syndrome; SSL, shoulder subluxation; VTE, venous thromboembolism; SHS, shoulder-hand syndrome; HO, heterotopic ossification; USI, urinary system infection; PS, pressure sores; CI, secondary cerebral infarction after stroke; CIA, cognitive impairment assessment; DOC, disturbance of consciousness; ED, emotional disorder; E/V impairment, eyesight/vision impairment; and HD, headache or dizziness.

Most cases (98.70%) in this study were identified as severe cases (Figure 3A). The incidence of epidural hematoma and diffuse axonal injury among females was significantly higher as compared to those among the males (*P* < 0.01, OR = 13.14; *P* < 0.01, OR = 6.30), while the incidence of intracranial hematoma (and brain herniation) among females was significantly lower as compared to that among the males (χ^2^ = 27.78, *P* < 0.01, OR = 1.38; χ^2^ = 7.40, *P* < 0.01, OR = 4.18).

The surgery rate among all the patients was 70.19%, with little gender difference (71.84% *vs.* 65.22%). Most of the surgeries were performed for the removal of hematoma using craniotomy (69.54% *vs.* 60%), followed by hematoma puncture and drainage (2.30% *vs.* 5.22%) (Figure 3B). Among all the patients, undergoing surgery, 59 cases (18.15%) went through a second surgical procedure, which included puncture and drainage of hematoma.

Hydrocephalus (46.84%), epilepsy (27.87%), and shoulder subluxation (12.64%) were the most common complications among males (Figure 3C). On the other hand, among females, hydrocephalus (45.22%), shoulder subluxation (19.13%), and disuse syndrome (16.52%) were the most common complications, while the incidence of epilepsy was slightly lower among them (15.65%).

Among the types of dysfunctions, both the males and females showed higher consistencies (Figure 3D). Cognitive impairment (66.09% *vs.* 62.61%), dyskinesia (61.21% *vs.* 70.43%), and language impairment (32.76% *vs.* 36.52%) were the most common types dysfunctions. Bilateral dyskinesia showed the highest consistency (31.03% *vs.* 32.17%).

### 3.2. GA-BP neural network model

#### 3.2.1. Construction of GA-BP model

Based on their correlations to the actual FMA, all the 11 prognostic-related factors were included in the prediction model (Table 1). After constructed the mode with 175 samples, the GA-BP neural network was built and the code can be found in https://github.com/swlmed/swlmed-public. Pearson correlation between predicted and actual values of the model was *R*^2^ = 0.97 and both of these values were shown in Supplementary Figure 2. The weight of every features to neurons and neurons to output listed in Table 2.

**TABLE 2:** Weights of features to neurons (w1) and neurons to output (w2).

Neuron	Course	Gender	Rehabilitation	Epilepsy	Hydrocephalus	Aphasia	Tracheotomy	Complications	Hands function	FMB	FMA	W2
1	0.13	0.36	-0.24	-0.5	-0.26	-0.26	0.05	-0.57	-0.29	-0.02	0.1	0.16
2	0.42	0.66	-0.34	0.19	0.56	0.38	0.07	0.95	-0.64	-0.2	-0.29	-0.13
3	0.52	-0.58	-0.06	-0.33	0.08	-0.1	-0.69	0.4	-0.03	-0.46	-0.23	-0.37
4	0.37	-0.64	0.25	0.27	-0.7	-0.16	0.3	0.78	0.32	-0.29	-0.43	-0.16
5	-0.62	0.56	0.69	-0.38	0.31	-0.61	0.2	-0.04	0.34	-0.27	-0.6	0.38
6	0.13	-0.89	-0.09	-0.04	-0.65	-0.09	-0.04	-0.16	0.44	0.58	-0.79	0.76
7	0.07	0.29	0.14	-0.06	0.43	-0.62	-0.34	0.28	0.01	-0.43	-0.24	0.26
8	0.01	-0.59	0.17	0.63	-0.45	-0.3	0.47	0.53	0.01	0.69	0.22	0.42
9	-0.75	0.75	0.27	-0.55	0.37	-0.38	0.41	-0.12	-0.66	0.24	0	0.79
10	-0.03	-0.06	0.5	0.21	0.41	-0.25	0.48	-0.23	-0.37	0.56	-0.59	-0.04

#### 3.2.2. Performance of GA-BP model

**FIGURE 4:**
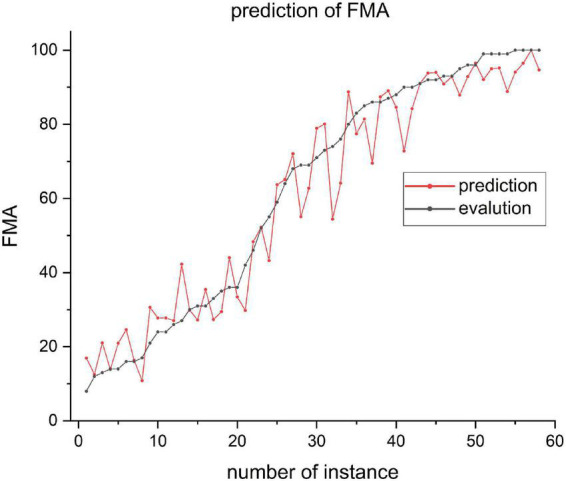
Predictive and actual FMA value of 58 patients.

**FIGURE 5:**
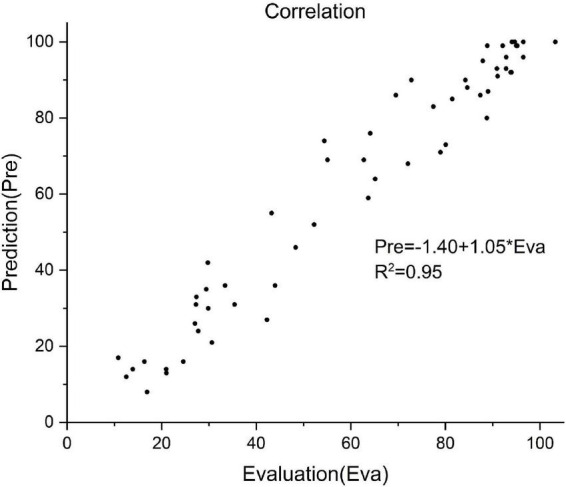
Correlation between the predictive and actual FMA value of 58 patients. Prediction value = –1.40 + 1.05 × actual value, *R*^2^ = 0.95.

Of all the 58 test patients, the predicted and actual FMA values are shown in Figure 4. Pearson correlation analysis was used to evaluate the differences between the predicted and actual FMA values (*R*^2^ = 0.95, *P* < 0.01, Figure 5).

## 4. Discussion

Backpropagation neural network is a multi-layer feed forward network trained by the error BP algorithm, proposed by Rumelhart et al. (1986). The BP network stores a large number of input-output pattern mapping correlations, describing the mapping correlation without the need for revealing the mathematical equations. Using the gradient descent method, it continuously adjusts the network through BP and minimizes the sum of squared errors of the network. Therefore, it also avoids the use of linear correlations between data. Numerous factors, affecting the degree of injury and prognosis of TBI patients, have unclear correlations among them. Currently, BP neural network is an easily applicable model (Rau et al., 2018).

Although mild TBIs account for the majority of the cases, in this study, moderate to severe TBIs accounted for the majority of the cases (98.70%). The reasons might include the lower hospitalization rate of the patients with mild TBI. Moreover, the patients with concussions mostly complaint about headaches, nervousness, inattention, forgetfulness, etc., which can be cured. The severely affected limb function is mainly manifested in the patients with moderate to severe TBI. Therefore, the improvement of motor function was mainly studied among these patients (Levin and Diaz-Arrastia, 2015; Hon et al., 2019; Silverberg et al., 2020).

In this study, correlation analysis was used to explore the prognostic factors. For this purpose, 11 prognostic-related indicators were investigated. In the current study, age was not included as a prognostic factor, as used in the previously reported mortality prediction models, affecting the survival rate of the TBI patients (Gang et al., 2019; Li et al., 2020). After involving the rehabilitation period, the focus of rehabilitation gradually changed from the central nervous system to the rehabilitation of the central-peripheral joint with the weakened effect of neuroplasticity, among which, age might play a less important role than that in the acute period. Unlike the previous prognostic prediction models, this study predicted continuous changes in the FMA, rather than categorizing the results, thereby showing relatively more refinement of the results. As compared to the real results, this prediction model had a satisfying accuracy rate (*R*^2^ = 0.95). The reliability of the results was obtained by the integration of training, validation, and test datasets.

In order to establish the TBI prediction model, ANN was used for predicting the prognosis and complications due to its flexibility and other advantages (DiRusso et al., 2000; Rughani et al., 2010; Hannah et al., 2020; Wang et al., 2021). ANN was superior to the TRISS model in terms of accurate mortality prediction (Matsuo et al., 2020). ANN could achieve higher accuracy than the CRASH and IMPACT models in terms of predicting mortality in patients with moderate to severe TBIs (Rau et al., 2018). ANN was more sensitive than the traditional models in predicting the mortality of the patients with severe cases (Yao et al., 2020), underwent surgery (Shi et al., 2013), mechanical ventilation (Abujaber et al., 2020a,b), septicemia (Kim et al., 2011), as well as in identifying the clinically relevant pediatric TBI (Hale et al., 2018).

The data-driven prediction models, such as CRASH and IMPACT, were based on the databases; as the database updated, their prediction accuracies improved (Hannah et al., 2020). This implied that the data-driven could be continuously improved with the refining of data. Therefore, for these models, the critical point is a database with sufficient data. Although, China collects the TBI information through various systems, such as the Nationally Representative Disease Monitoring Point System (DSP), Hospital Quality Monitoring System (HQMS), and National Injury Monitoring System (NISS), a uniform data system for medical rehabilitation (UDMSR) for the collection of data information in a unified way is currently lacking (Granger et al., 2010; Oyesanya et al., 2021). In this study, a database, containing the rehabilitation information of 463 patients, was established. The outcome measure of this study was FMA, which is one of the most commonly used indices of motor function. In the end, only 233 patients were included in this study. The information in the database was inefficient, which might be due to the following reasons. First, the patients under rehabilitation might have more comorbidities; therefore, numerous factors might affect the rehabilitation treatment. Second, the patients came from different areas in China. Therefore, it was difficult to follow up with the patients after discharge for the follow-up assessment.

This study started with the admission of patients with TBI to a rehabilitation hospital and used the functional assessment as the baseline. The characteristics of TBI were elucidated and the prediction model based on FMA was established to predict the outcome of motor dysfunction. The prediction model had low requirements, strong operation ability, and easy promotion. It had high practicability for the hospitals with fewer research resources and might serve as a reference in the risk and prognosis assessment as well as guiding the clinical decision-making.

## 5. Limitations

The first limitation of this study was the small sample size obtained from a single medical center. This study lacked the indicators, which directly reflected the severity of TBI in the patients. Therefore, this might have affected the results. Second, the model could not clearly explain which factor was listed at the top. Finally, this study assumed unified rehabilitation therapies for all the patients and did not consider their differences in them.

In addition to the above-mentioned shortcomings, this study used neural network and regression models to predict the prognosis of motor function in patients with TBI and provided novel ideas for the evaluation of the patients with TBI.

## Data availability statement

The original contributions presented in this study are included in the article/Supplementary material, further inquiries can be directed to the corresponding authors.

## Author contributions

HD contributed to the study design, literature search, figures, data extraction, data analysis, and writing. ZT and WS contributed to the figures, data extraction, data interpretation, and writing. HZ and SY contributed to the study design and data interpretation. All authors contributed to the article and approved the submitted version.
