# Corrigendum: Prediction of motor function in patients with traumatic brain injury using genetic algorithms modified back propagation neural network: a data-based study

**DOI:** 10.3389/fnins.2023.1208118

**Published:** 2023-05-03

**Authors:** Hui Dang, Wenlong Su, Zhiqing Tang, Shouwei Yue, Hao Zhang

**Affiliations:** ^1^Cheeloo College of Medicine, Shandong University, Jinan, Shandong, China; ^2^China Rehabilitation Research Center, Beijing, China; ^3^School of Health and Life Sciences, University of Health and Rehabilitation Sciences, Qingdao, Shandong, China

**Keywords:** TBI, back propagation neural network, prediction model, FMA, database

In the published article, there was an error in affiliation with duplicate affiliation 2 and 4, delete 4. Instead of “Wenlong Su^3, 4^,” it should be “Wenlong Su^2, 3^,” instead of “Zhiqing Tang^4^,” it should be “Zhiqing Tang^2^,” instead of “Hao Zhang^1, 3, 4^,” it should be “Hao Zhang^2, 1, 3^”.

There was an error in affiliation 1. Instead of “Qilu Hospital, Cheeloo College of Medicine, Shandong University, Jinan, Shandong, China”, it should be “Cheeloo College of Medicine, Shandong University, Jinan, Shandong, China”.

The authors apologize for this error and state that this does not change the scientific conclusions of the article in any way. The original article has been updated.

