# Supplementary material for: Prediction of motor function in patients with traumatic brain injury using genetic algorithms modified back propagation neural network: a data-based study
Source: Front Neurosci. 2023 Jan 19;16:1031712. doi: 10.3389/fnins.2022.1031712 (PMC9892718; doi:10.3389/fnins.2022.1031712)
Supplement: Supplementary file 1 [file Data_Sheet_1.docx]

Supplementary Material

|  | Overall(n=463) | Male(n=348) | Female(n=115) |
| --- | --- | --- | --- |
| **Course of disease(d)**  <30  30~90  90~180  180~365  >365 | 272.61±550.29  34(7.34%)  129(27.86%)  143(30.89%)  86(18.57%)  71(15.33%) | 304.63±618.94  24(6.90%)  100(28.74%)  100(28.74%)  62(17.82%)  62(17.82%) | 175.74±220.35  10(8.70%)  29(25.22%)  43(37.39%)  24(20.87%)  9(7.83%) |
| **Age(y)**  <18  18~30  30~45  45~60  60~75  >75 | 42.15±17.38  54(11.66%)  72(15.55%)  132(28.51%)  121(26.13%)  73(15.77%)  11(2.38%) | 42.62±17.40  40(11.49%)  50(14.37%)  98(28.16%)  102(29.31%)  47(13.51%)  11(3.16%) | 40.70±17.33  14(12.17%)  22(19.13%)  34(29.57%)  19(16.52%)  26(22.61%)  0(0.00%) |
| **Hospital stay(d)**  <15  15~30  30~45  45~60  60~90  >90 | 45.68±34.03  32(6.91%)  82(17.71%)  192(41.47%)  54(11.66%)  70(15.12%)  33(7.13%) | 46.54±36.91  24(6.90%)  64(18.39%)  139(39.94%)  43(12.36%)  51(14.66%)  27(7.76%) | 43.10±23.14  8(6.96%)  18(15.65%)  53(46.09%)  11(9.57%)  19(16.52%)  6(5.22%) |
| **Marital status**  unmarried  married  widowed  divorced | 127(27.43%)  329(71.06%)  2(0.43%)  5(1.08%) | 94(27.01%)  248(71.26%)  1(0.29%)  5(1.44%) | 33(28.70%)  81(70.43%)  1(0.87%)  0(0.00%) |
| **Personal history (smoke/drinking)**  none  smoke or drinking  smoke and drinking | 344(74.30%)  73(15.77%)  46(9.94%) | 235(67.53%)  68(19.54%)  45(12.93%) | 109(94.78%)  5(4.35%)  1(0.87%) |
| **Medical history** hypertension  diabetes  CVD(cere- and car-)*  TBI  more than one  none | 58(12.52%)  17(3.67%)  1(0.22%)  1(0.22%)  42(9.07%)  344(74.30%) | 54(15.52%)  13(3.74%)  1(0.29%)  1(0.29%)  33(9.48%)  246(70.69%) | 4(3.48%)  4(3.48%)  0(0.00%)  0(0.00%)  9(7.83%)  98(85.22%) |

The general information of 463 patients. *cere-, cerebrovascular disease; car-, cardiovascular disease.


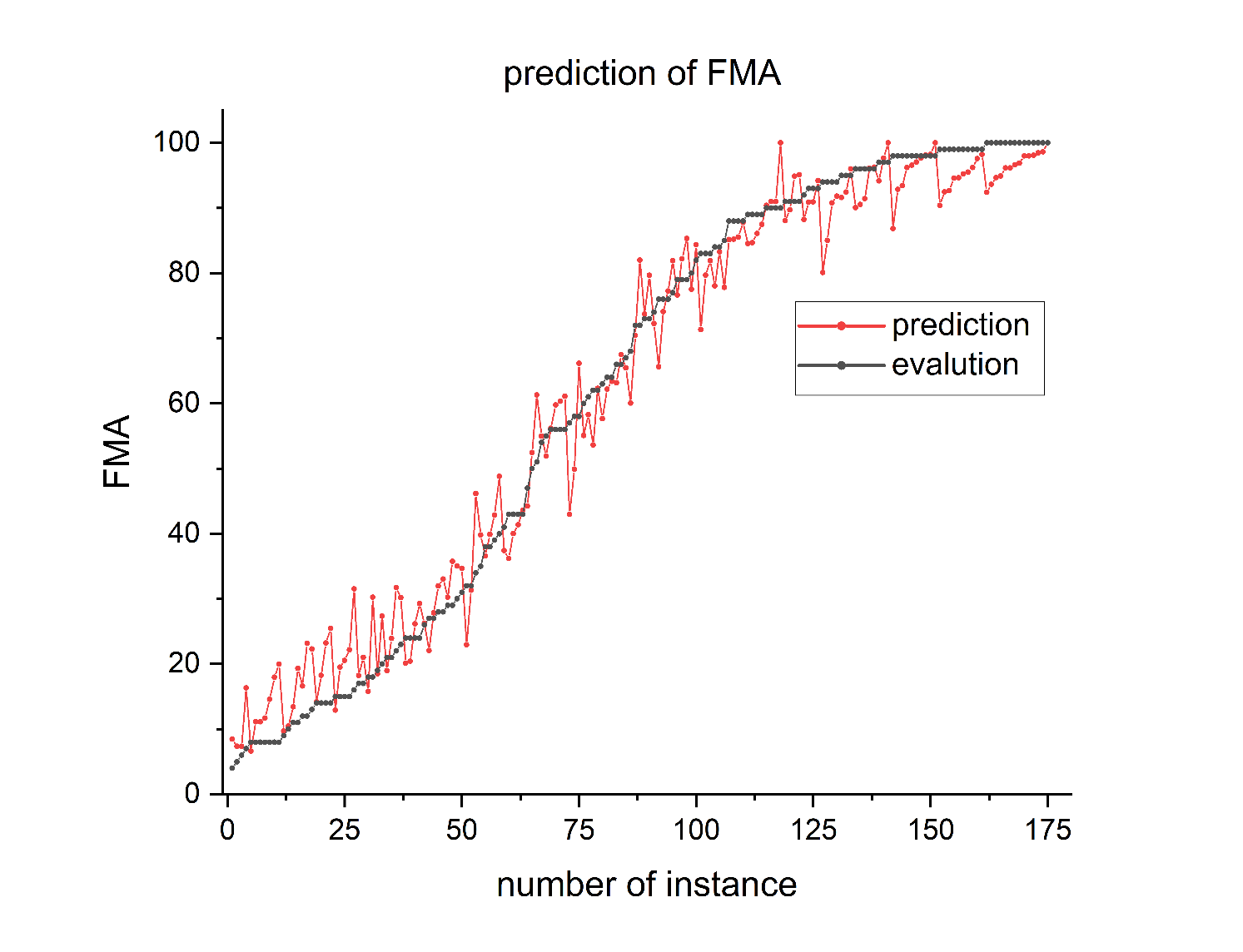


Figure 1. Predictive and actual FMA value of 175 patients.


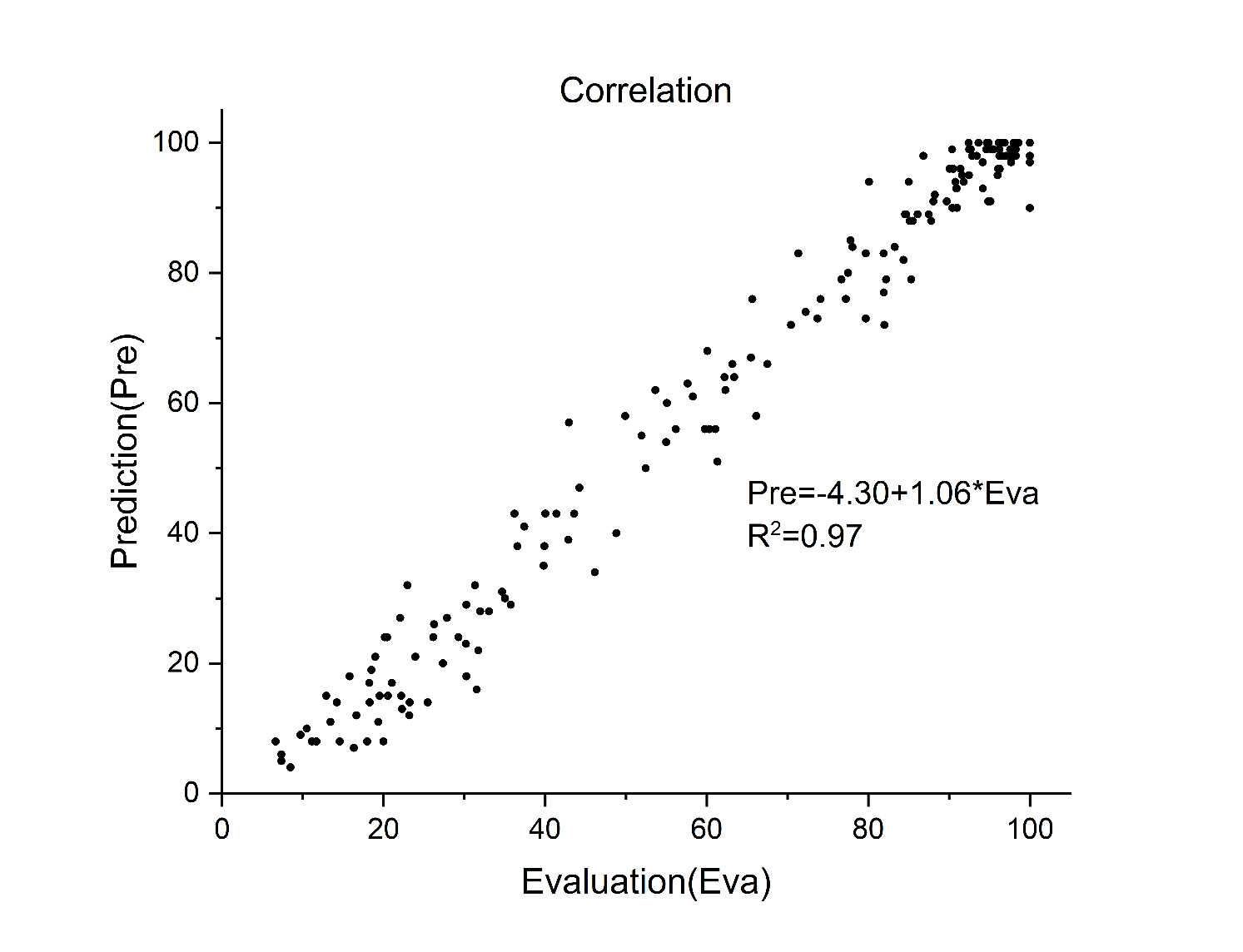


Figure 2. Correlation between the predictive and actual FMA value of all the patients. Prediction value = -4.30 + 1.06*actual value, R2 = 0.97
